# Clinical evaluation of postoperative nausea and vomiting after cleft lip and/or palate surgery in pediatric patients. Part 2: evaluation of preventive administration of droperidol in combination with dexamethasone

**DOI:** 10.20407/fmj.2018-008

**Published:** 2019-02-06

**Authors:** Takako Aizawa, Koji Satoh, Yoshikazu Kobayashi, Taroh Okui, Yohsuke Takehara

**Affiliations:** Department of Dental and Oral & Maxillofacial Surgery, School of Medicine, Fujita Health University, Toyoake, Aichi, Japan

**Keywords:** Pediatric patient, Cleft lip and/or palate, Postoperative nausea and vomiting, Preventive care

## Abstract

**Objectives:**

This study aimed to evaluate the effect of preventive administration of the combination of droperidol and dexamethasone on lowering the risk for postoperative nausea and vomiting (PONV) after cleft-related surgery in pediatric patients.

**Methods:**

Preventive care consisted of a single dose of droperidol (0.025 mg/kg) and dexamethasone (0.06 mg/kg), which were administered at the end of surgery. The effect of preventive administration was evaluated in a sample group of 58 patients aged ≥3 years who underwent cleft-related surgery. Thirty patients received preventive administration (prevention group) and 28 patients did not (comparative group). The following outcome variables were evaluated between the groups: sex, age, body weight at the time of surgery, and duration of anesthesia. The presence or absence of PONV was the primary outcome and other variables were considered as explanatory variables.

**Results:**

The incidence rate of PONV was 20% (6/30) in the prevention group and 28.6% (8/28) in the comparative group, with no significant difference between the groups (p=0.45). In multiple logistic regression analysis, sex was the only explanatory factor of PONV, with a higher risk in girls than in boys (odds ratio, 6.20; 95% confidence interval, 1.65–27.63; p=0.01).

**Conclusions:**

The incidence rate of PONV is 20% with preventive care of droperidol and dexamethasone administration, but this rate is not different from that without this combination. Sex is a risk factor for PONV. Further studies are required to validate our results.

## Introduction

Postoperative nausea and vomiting (PONV) is a common postoperative complication of surgery under general anesthesia in children. Although PONV rarely occurs in children younger than 3 years, the frequency of PONV in children aged 3 years or older is twice as high as that in adults. Therefore, the risk of PONV is considered to be high in children, especially after undergoing particular surgical procedures, such as tonsillectomy.^1,2^ However, the occurrence of PONV following cleft-related surgery has not been specifically addressed. In a previous study, we reported an incidence rate of PONV in children who had undergone cleft-related surgery as 21.7%.^3^ This study aimed to evaluate the effect of preventive care in lowering the incidence rate of PONV after cleft-related surgery in children aged ≥3 years.

## Methods

### Patients

Our retrospective analysis included data of 58 patients aged ≥3 years who had undergone cleft-related surgery (hard palatoplasty in a two-stage palatoplasty and secondary bone grafting) under general anesthesia at our institution. Patients were classified into two groups for analysis as follows: those who received preventive administration (prevention group; 30 patients, treated between September 2016 and February 2017) and those who did not (comparative group, 28 patients, treated between May 2016 and August 2016).

### Methods

Preventive care was based on the consensus guidelines for managing PONV from the Society for Ambulatory Anesthesiology (SAMBA).^4^ Although the combination of a 5-hydroxytryptamine (5-HT_3_) receptor antagonist and dexamethasone is recommended as first-line therapy in the SAMBA guideline,^4^ 5-HT_3_ receptor antagonists are not authorized by health insurance in Japan for preventing PONV. Therefore, we selected combination administration of 0.025 mg/kg of droperidol and 0.06 mg/kg of dexamethasone at the end of the surgery in this study.

Information on the following variables was retrieved from the medical records for analysis: sex, age at the time of surgery, body weight at the time of surgery, and duration of anesthesia. These variables were compared between patients with and those without PONV using the chi-squared or Mann–Whitney U test as appropriate for the data. The level of statistical significance was set at p=0.05. Multiple logistic regression was performed to identify risk factors for PONV. The presence or absence of PONV was the dependent variable and the remaining variables were explanatory factors. These variables were entered into a model using the stepwise method, with p=0.2 as the criterion for inclusion or exclusion of a factor. Statistical analyses were performed using JMP-11 statistical software (SAS, Tokyo, Japan).

### Ethical Considerations

This study was conducted with adherence to standard clinical practices following approval from the Institutional Review Board of Fujita Health University (No. HM17-100).

## Results

The distribution of sex was comparable between the prevention (boys, 20; girls, 10) and comparative (boys, 17; girls, 11) groups (chi-squared test, p=0.64). The distribution of the explanatory variables is shown in Table 1. No significant between-group differences were observed.

The incidence rate of PONV was 20% in the prevention group (6/30 cases) and 28.6% in the comparative group (8/28 cases), with no significant between-group difference (chi-squared test, p=0.45; Table 1).

In multiple logistic regression analysis, only sex was retained as an independent risk factor of PONV, with a higher incidence in girls than in boys (odds ratio, 6.20; 95% confidence interval, 1.65–27.63, p=0.01).

## Discussion

In 2014, the SAMBA published guidelines for assessment of the risk for PONV, as well as strategies for its prevention and treatment, underlining the importance of preventive treatment.^4^ Eberhart et al.^5^ identified an age ≥3 years, a duration of surgery >30 min, surgery for correction of strasbimus, and a prior history of PONV as risk factors for PONV. They also found that the presence of three risk factors was associated with a 50% risk of PONV. In our previous study, we reported an incidence rate of PONV in children who had undergone all cleft-related surgery as 21.7%.^3^ We also found that sex and duration of surgery were risk factors in multiple logistic regression analysis using the stepwise method. Sex was an independent factor because we thought that the clinical significance of the surgery time was low. Because these factors are not modifiable, preventive care through administration of medication in accordance with the SAMBA guidelines is the only viable option to lower the incidence of PONV.^3^

The vomiting center of the human brain is located in the bulbar reticular formation, with afferent stimulation via five different pathways. These pathways are as follows: vagal nerve afference, activated by 5-HT_3_ receptors present in the pharynx and upper gastrointestinal tract; vestibular-labyrinthine afference, via the eighth cranial nerve; visual afference; cerebral limbic afference, activated by psychological factors; and afference from the chemoreceptor trigger zone of the medulla.^6^ Therefore, a multifactorial approach is required for prevention of PONV, with administration of more than one antiemetic drug recommended.^4,7^

The SAMBA guidelines provide the following evidence-based strategies for prevention of PONV in pediatrics: a single dose of ondansetron, droperidol, and dexamethasone (level of evidence, A1) or a combination of ondansetron with dexamethasone or droperidol (level of evidence, A1). Moreover, for pediatric patients at high risk for PONV, a combination of a 5-HT_3_ receptor antagonist and dexamethasone is recommended as first-line therapy,^4^ in the absence of contraindications. With regard to preventive care for PONV in patients undergoing cleft-related surgery, we conducted a literature search of the PubMed and Ichushi Web databases using the following search terms: “child”, “cleft palate”, “postoperative vomiting”, and “postoperative nausea and vomiting”. Our search did not identify any study between 2000 and 2017 regarding preventive strategies for PONV in this surgical population. In our search, we took into account that cleft-related surgery is considered in the field of maxillofacial surgery, with the region located near the palatine tonsils. For children undergoing tonsillectomy, dexamethasone and anti-serotonergic agents appear to be the most effective agents for prophylaxis for PONV.^7^ The 5-HT_3_ receptor antagonists selectively inhibit binding of serotonin, both centrally and peripherally, with droperidol acting centrally as a dopamine antagonist. Dexamethasone, which has strong antiemetic action, is added as a standard therapy for cancer chemotherapy, despite an unclear mechanism of action.^8^

In a previous study, we considered that the reason why PONV is higher in children aged ≥3 years is that younger patients often cannot describe feelings of retching or nausea and that mental factors are affected more strongly with maturation.^3^ Therefore, in this study, we only included children aged ≥3 years, and applied combination administration of a single dose of droperidol (0.025 mg/kg) and dexamethasone (0.06 mg/kg) at the end of surgery. We could not select first-line therapy in the SAMBA guideline because 5-HT_3_ receptor antagonists are not authorized by health insurance in Japan for prevention of PONV. The incidence of PONV was 20% in the prevention group and 28.6% in the comparative group, but the between-group difference was not significant. In a systematic review study, Bolton et al.^7^ evaluated 557 children who underwent tonsillectomy/adenoidectomy. They found that the incidence of POV was sufficiently high (up to 70%), and that ondansetron was more effective than metoclopramide in preventing POV. They also found that 5-HT_3_ antagonists and dexamethasone were the most effective prophylactic antiemetics with insufficient evidence for the efficacy of droperidol. These authors suggested that prophylaxis with a combination of a 5-HT_3_ antagonist and a steroid should be administered for most pediatric patients at high risk for POV unless there is a contraindication.

Apfel et al.^8^ reported a randomized, controlled trial on 4123 patients who were randomly assigned to combinations of three prophylactic antiemetic interventions. In their study, the incidence of PONV varied considerably among various sites of surgery. Overall, 34% of patients experienced PONV, and ondansetron, dexamethasone, and droperidol each reduced the incidence of PONV by 26%.

In the present study, the incidence of PONV was reduced by 29% with preventive administration using the combination of droperidol and dexamethasone for cleft-related surgery in pediatric patients. Multiple regression analysis showed that sex was the only independent risk factor for PONV, with the risk of PONV significantly greater in girls than in boys. Therefore, provision of preventive care for PONV should be considered at least for girls undergoing cleft-related surgery. Importantly, our combined use of droperidol and dexamethasone was not effective in suppressing PONV after cleft-related surgery. In a future study, we will consider the use of a 5-HT_3_ receptor antagonist, despite the current health insurance coverage.

This study has some limitations. Statistical power may have been limited because of the small amount of available data. The birth prevalence of cleft lip and/or palate is approximately 1:500 in Japan (World Health Organization, 2001).^9^ Therefore, increasing the size of the cohort in a single institution is difficult. In the future, a clinical study with a sufficient number of cases should be performed as a collaborative research project.

In conclusion, the incidence rate of PONV is 20% after administration of droperidol in combination with dexamethasone, but this finding is not significant. Sex is a risk factor for PONV.

## Figures and Tables

**Table1:** Comparison of survey items between the prevention and comparative groups

Survey items		Prevention group	Comparative group	p value
PONV	Positive	6 (20.0%)	8 (28.6%)	
	Negative	24 (80.0%)	20 (71.4%)	0.45
Sex	Male	20	17	
	Female	10	11	0.64
Age at the time of surgery, mo		109.5 (68.3–134.0)	112.0 (80.0–132.8)	0.55
Body weight at the time of surgery, kg		27.7 (17.3–36.6)	26.1 (20.3–31.9)	0.83
Duration of anesthesia, min		150.0 (131.5–182.0)	144.0 (135.3–153.5)	0.21

Data on age at the time of surgery, body weight at the time of surgery, and duration of anesthesia are presented as the median value (interquartile range).
